# Body Mass Index and Breast and Cervical Cancer Screening

**DOI:** 10.1089/whr.2021.0062

**Published:** 2022-05-09

**Authors:** Elfreda Samman, Rahma Mkuu, Xiaoying Zhang, Shelby Scummings, James Burdine

**Affiliations:** ^1^ Department of Health Promotion and Community Health Sciences, School of Public Health, Texas A&M University, College Station, Texas, USA.; ^2^ Department of Health Outcomes and Biomedical Informatics, College of Medicine, University of Florida Gainesville, Florida, USA.; ^3^ Department of Health and Kinesiology, College of Education & Human Development, Texas A&M University, USA.; ^4^ Department of Statistics, College of Science, Texas A&M University, USA.

**Keywords:** body mass index, breast cancer, cervical cancer, women, preventative screening

## Abstract

**Background:**

Breast and cervical cancer screening are responsible for dramatically reducing cancer deaths. Overweight and obesity are associated with deleterious health outcomes, including increased risk of developing cancer. This study adds to the existing literature examining the association of having overweight and obesity and receipt of breast or cervical cancer screening.

**Methods:**

Using the 2013 Brazos Valley Community Health Needs Assessment, we examined the association between body mass index (BMI) and receipt of breast or cervical cancer screening among women meeting age recommendations for breast cancer and cervical cancer screening (*n* = 1979 and *n* = 2040), respectively. We used SPSS 22 statistical software for descriptive and logistic regression analysis.

**Results:**

Overall, 26.6% of women missed the breast cancer screening guidelines, and 13.3% missed the cervical cancer screening guidelines. BMI had a weak association with missing cervical cancer screenings (odds ratio [OR] = 1.02; confidence interval [CI] = 1.01–1.04), but no association with missing breast cancer screenings (OR = 1.01; CI = 0.99–1.03). Higher age, race (non-White), rural area, no health insurance, smoking, and delayed health care were associated with missing breast cancer screenings. Higher age, marital status (single), lower education, no health insurance, smoking, and delayed health care were associated with missing cervical cancer screening. Further research is needed to better understand the association using larger, more diverse samples.

## Introduction

Breast and cervical cancer are among the four most common causes of cancer death among women worldwide.^1–4^ In the United States, cancer is responsible for 21.6% of adult female deaths each year, making it the second leading cause of death for women in the country.^3^ Among all cancers, breast cancer continues to be the leading cause of cancer death for women in the United States, while cervical cancer, despite being preventable, continues to be a public health challenge.^5,6^

Advancements in preventative care, such as screenings, have resulted in early detection and a reduction in cancer deaths.^7^ Among all cancer deaths in the United States, 3%–35% of premature deaths could be prevented through early screening.^8^ For example, cervical cancer used to be the leading cause of cancer death for women, but remarkable reductions in the number of deaths are directly associated with increased cervical cancer screening (Pap testing).^6,9^

The U.S. Preventative Services Task Force (USPSTF) recommends screening with cervical cytology alone for all women between 21 and 29 years of age every 3 years. For women 30–65 years old, the USPSTF recommends screening with cervical cytology alone every 3 years and high-risk human papillomavirus (hrHPV) testing alone every 5 years, or contesting with hrHPV and cytology every 5 years.^10^ For breast cancer, biennial mammography screening is recommended for women 50–74 years old.^11^

In addition to screening, maintaining a healthy body weight is increasingly being acknowledged as a preventative method for cancer.^12,13^ Body mass index (BMI) is a measure of a person's weight in kilograms by their height in meters.^14^ A BMI of 25.0–29.9 is considered overweight while 30.0 and above is considered obesity.^14^ The growing cancer epidemic has been linked to the increasing overweight and obesity epidemic.^15,16^ Global estimates show that 39% of adults have overweight and 13% have obesity, and women are the group with the highest prevalence of these conditions.^1^

In the United States, over 70% of adults 20 years of age and above are overweight or obese; 37.9% have obesity and 7.7% have extreme obesity.^17^ Similar to global trends, women in the United States are more likely have obesity than men (40.5% vs. 35.2%).^17^ Among all cancers, one in five diagnosed are associated with obesity and its related risk behaviors, such as low physical activity, poor nutrition, and excess alcohol consumption.^18^

It is well established that obesity is a risk factor for cancer among women.^19,20^ The risk of breast cancer increases by 12% for every 5-unit increase in BMI; moreover, women who have obesity have a 20%–40% increased risk of breast cancer compared with women who do not have obesity.^13,21,22^ The association between obesity and breast cancer is particularly concerning since patients with obesity who are diagnosed with cancer are more likely to present with later stages of cancer and larger tumors and more aggressive forms of cancer than their counterparts with normal weight.^3,23,24^ In addition, women who have obesity have higher breast cancer mortality rates.^25^ Obesity is also associated with an increased cervical cancer risk.^26,27^

The increased risk for cervical and breast cancer among women who have obesity or are overweight has raised the question as to whether there is a relationship between body weight and screening rates for breast and cervical cancer.^28,29^ Some evidence exists that increased BMI is associated with decreased breast/cervical screening.^30–32^ However, most of those studies were conducted outside of the United States.^33–36^ Among studies on this topic in the United States, including systematic reviews, the majority are more than 10 years old.^29,31,37,38^ Furthermore, the more recent studies in the United States do not focus on the general female population but on specific groups, such as women who are veterans.^39,40^

The purpose of this study was to examine the association between BMI and breast and cervical cancer screening guideline concordance. This study adds to the scarce literature on community-based population studies examining the association between BMI and receipt of breast and cervical cancer screening in the United States.

## Methods

### Data source

Data from the 2013 Brazos Valley Health Assessment (BVHA), a survey, were used for this study. The BVHA was administered in seven counties in the Brazos Valley Region located in south-central Texas. BVHA participants were recruited through random-digit-dialed telephone screening. Prospective participants were mailed a survey booklet, cover letter, incentive, and postage-paid envelope. The study was reviewed and approved by the Texas A&M University Institutional Review Board. Details on the methodology for recruitment can be found in a previous study.^41^

### Study variables

#### Dependent variable

The dependent variable was whether the respondent had missed a breast cancer or cervical cancer screening as recommended by USPSTF in 2013 when the survey was administered. The USPSTF recommends screening with cervical cytology alone for all women between 21 and 29 years of age every 3 years. For women 30–65 years old, the USPSTF recommends screening with cervical cytology alone every 3 years and hrHPV testing alone every 5 years, or contesting with hrHPV and cytology every 5 years.^10^

Only women who were within the recommended screening age range for breast cancer screening (50–75 years, *n* = 1979) and for cervical cancer screening (21–65 years, *n* = 2040) were included for the analyses. This question in the survey asked the last time in years they had a mammogram or pap smear.

#### Independent variables

BMI was a continuous variable calculated using self-reported weight and height. Furthermore, the following covariates were added to our initial analyses: age was a continuous variable; race was a binary variable coded as White or non-White as the sample lacked sufficient number of underrepresented racial/ethnic groups for comparison.

Marital status was a categorical variable coded as married, widowed, divorced/separated, never married, or living with a partner; education was a categorical variable coded as less than high school, high school, college degree, or greater than a college degree; geographical location was a binary variable coded as rural or urban; health insurance was a binary variable coded as yes or no; smoker was a binary variable coded as yes or no; and delay in health care was binary and coded as a yes or no.

### Statistical analyses

All statistical analyses were conducted using SPSS version 22. Binary logistic regression was used for each outcome variable. Descriptive statistics were calculated for selected sample characteristics. Two binary regression models were fitted to determine the association between BMI and breast cancer screening or BMI and cervical cancer screening. For each binary logistic regression, the backward elimination method was used, and the best fit model was selected. Results were reported as odds ratios (ORs) with a 95% confidence interval (CI); a *p*-value <0.05 was considered statistically significant.

## Results

Sample characteristics are presented in Table 1. The average BMI of our sample was 28.3. The average age was about 56 years, the majority (82.7%) were White, 76.6% were married, 70.7% had higher than a high school diploma, 66.2% lived in urban areas, 89.8% were nonsmokers, 92.3% had health insurance, and 63.8% did not delay seeking health care services. Among women 21–65 years of age, 13.3% missed the recommended cervical cancer screening guidelines, and among women 50–74 years of age, 26.6% missed the recommended breast cancer screening guidelines.

**Table 1.** **Sample Characteristics, Women Age 21–74 (*n*** **= 3014)**

**Characteristic**	**Mean (SD) or %**
BMI	28.3 (7.20)
Obese/overweight	38.9
Age	55.6 (11.4)
Race	
White	82.7
Non-White	17.3
Marital status	
Married	76.6
Widowed	8.0
Divorced/separated	10.1
Never married	3.6
Living with partner	1.8
Education	
<High school	6.4
High school	22.9
>High school	70.7
Geographical area	
Rural	33.8
Urban	66.2
Smoking	
Yes	10.2
No	89.8
Health insurance	
Yes	92.3
No	7.7
Delay in health care	
Yes	36.2
No	63.8
Missed breast cancer screening recommendations	26.6
Missed cervical cancer screening recommendations	13.3

BMI, body mass index; SD, standard deviation.

Table 2 shows the results of the regression analyses of the association between breast and cervical cancer screenings and predictor variables. The ORs are reported for each variable.

**Table 2.** Odds Ratios for Missing Breast and Cervical Cancer Screening

**Characteristic**	**Missed breast cancer screening** ***n* = 1979**		**Missed cervical cancer screening** ***n* = 2040**	
	**OR**	**95% CI**	**OR**	**95% CI**
BMI	1.01	0.99–1.03	1.02^*^	1.01–1.04
Age	1.03^*^	1.01–1.05	1.04^*^	1.02–1.06
Race^a^				
Non-White	0.86	0.60–1.24	0.98	0.67–1.43
Marital status^b^				
Single	1.28	0.63–2.58	3.88^*^	2.18–6.89
Divorced/separated	1.12	0.77–1.63	1.38	0.89–2.15
Widowed	0.96	0.64–1.43	1.37	0.79–2.37
Living with partner	1.66	0.60–4.52	0.80	0.29–2.20
Education^c^				
High school	1.24	0.70–2.20	0.78	0.43–1.40
>High school	1.44	0.81–2.45	0.63	0.36–1.10
Geographical area^d^				
Rural	1.51^*^	1.18–1.93	1.25	0.92–1.70
Health insurance^e^				
Yes	0.22^*^	0.14–0.34	0.26^*^	0.17–0.38
Smoker^f^				
Yes	2.36^*^	1.64–3.38	2.24^*^	1.51–3.33
Delay in health care^g^				
Yes	2.39^*^	1.84–3.10	1.74^*^	1.28–2.37

^a^ Reference = white.

^b^ Reference = married.

^c^ Reference = less than high school.

^d^ Reference = urban.

^e^ Reference = no (health insurance).

^f^ Reference = nonsmoker.

^g^ Reference = no (delayed health care).

^*^ Significant at *p* < 0.05.

CI, confidence interval; OR, odds ratio.

### Breast cancer screening

Based on the binary logistic regression model, BMI was not a significant predictor of missing breast cancer screening (Tables 2 and 3). Several other covariates—age, race, geographical area, health insurance, smoking status, and health care-seeking behavior—were important predictors of breast cancer screening. In addition, non-Whites were less likely than Whites to miss breast cancer screening (OR = 0.86; CI = 0.60–1.24), whereas women living in rural areas were more likely to miss breast cancer screening than women in urban areas (OR = 1.51; CI = 1.18–1.93). Women with no health insurance were less likely to miss breast cancer screening than women who had no health insurance (OR = 0.22; CI = 0.14–0.34), and smokers were more likely to miss breast cancer screening than nonsmokers (OR = 2.36; CI = 1.64–3.38).

**Table 3.** Odds Ratios for Missing Breast and Cervical Cancer Screening by Body Mass Index Categories

**Characteristic**	**Missed breast cancer screening** ***n* = 1979**		**Missed cervical cancer screening** ***n* = 2040**	
	**OR**	**95% CI**	**OR**	**95% CI**
BMI^a^				
Underweight	1.63	0.64–4.09	0.81	0.24–2.75
Overweight	0.94	0.70–1.29	0.99	0.67–1.47
Obesity class I	1.03	0.72–1.48	1.28	0.83–1.99
Obesity class II	0.86	0.54–1.34	1.43	0.87–2.34
Obesity class III	1.53	0.94–2.51	1.66	0.95–2.88
Age	1.03^*^	1.01–1.05	1.04^*^	1.02–1.06
Race^b^				
Non-White	0.85	0.59–1.22	0.98	0.67–1.45
Marital status^c^				
Single	1.22	0.61–2.45	3.98^*^	2.24–7.07
Divorced/separated	1.13	0.78–1.64	1.37	0.88–2.14
Widowed	0.92	0.61–1.38	1.37	0.78–2.39
Living with partner	1.63	0.60–4.44	0.82	0.29–2.25
Education^d^				
High school	1.20	0.68–2.13	0.80	0.45–1.43
More than high school	1.36	0.79–2.37	0.63	0.36–1.15
Geographical area^e^				
Rural	1.52^*^	1.19–1.96	1.25	0.91–1.69
Health insurance^f^				
Yes	0.22^*^	0.14–0.34	0.26^*^	0.17–0.38
Smoker^g^				
Yes	2.28^*^	1.58–3.27	2.22^*^	1.49–3.30
Delay in health care^h^				
Yes	2.39^*^	1.84–3.11	1.74^*^	1.27–2.37

^a^ Reference = normal/healthy.

^b^ Reference = white.

^c^ Reference = married.

^d^ Reference = less than high school.

^e^ Reference = urban.

^f^ Reference = no (health insurance).

^g^ Reference = nonsmoker.

^h^ Reference = no (delayed health care).

^*^ Significant at *p* < 0.05.

Women who delayed seeking health care were also more likely to miss breast cancer screening (OR = 2.39; CI = 1.84–3.10) than women who did not delay. Marital status was not a significant predictor of missing breast cancer screening.

### Cervical cancer screening

Based on our binary logistic regression model, as shown in Table 2, BMI was a significant, but weak predictor of missing cervical cancer screening. Specifically, with a one unit increase in BMI (OR = 1.02; CI = 1.01–1.04), the respondent is 2% more likely to miss their cervical cancer screening. Also, with a 10 U increase in BMI, they are 20% more likely to miss their cervical cancer screening.

However, when BMI was categorized, there was no significant association with missing cervical cancer screening (Table 3). The covariates of age, marital status, smoking status, health insurance, and health care-seeking behavior were also significant predictors of missing cervical cancer screening. In terms of age, for every 1-year increase in age, there is a 4% likelihood of she will have missed a cervical cancer screening (OR = 1.04; CI = 1.02–1.04). With regard to marital status, single women were more likely to miss cervical cancer screening than married women (OR = 3.88; CI = 2.18–6.89).

Moreover, women with health insurance were significantly less likely to miss cervical cancer screening (OR = 0.26; CI = 0.17–0.38) than were women who had no health insurance. Finally, smokers (OR = 2.24; CI = 1.51–3.33) and those who delayed seeking health care (OR = 1.74; CI = 1.28–2.37) were more likely to miss their cervical cancer screening than nonsmokers and those who did not delay. Race and geographical area were not significant predictors of missing cervical cancer screening (Table 2).

## Discussion

We sought to explore whether BMI was associated with breast and cervical cancer screening among a community sample of women in Texas. Our findings indicated a weak association between an increase in BMI and missing cervical cancer screening, and no association between BMI and missing breast cancer screening.

There are currently conflicting findings of the association between BMI and breast and cervical cancer screening. Contrary to our results, in a Canadian study, the prevalence of cervical cancer screening in women was significantly less likely in women who were overweight/obese than those of normal weight.^42^ The findings are similar to U.S. data where marginally significant odds of having a routine cervical cancer screening decreased among women who were overweight/obese.^43^ In a population-based sample in a large population in the U.S. to assess whether receiving preventative services were related to BMI, women who were extremely obese (≥40.0) received lower mammograms and Paps overall.^44^

However, similar to our results, a U.S. study found that BMI was not significantly associated with mammography even though it was for breast exams and pap smears.^37^ One major difference between our study and previously mentioned studies is that BMI was divided into categories; therefore, we analyzed BMI as a continuous variable and in categories.

When divided into categories (Table 3), BMI was no longer a significant predictor of missing breast or cervical cancer, contrary to previously mentioned studies.^37,44^ However, it is important to note that categorizing variables may lead to loss of information, and statistical power is often reduced.^45^ There are currently limited recent studies highlighting the association between BMI and breast and cervical cancer screening.

Despite high cervical cancer mortality rates in some racial minorities,^3^ in this study, race was not associated with missed cervical cancer screenings; this finding may be due to previous efforts to increase screening among minority groups.^46–48^ However, women who are White were less likely to miss breast cancer screening than women who were non-White. Women who are Black may face anxiety in the health care setting due to the threat of negative stereotyping.^49^

These findings align with the results of several other studies. For instance, increased age has been associated with missed breast and cervical cancer screenings,^50,51^ as has a lack of health insurance.^52^ Furthermore, residents in rural areas are more likely to miss breast cancer screenings, and being single is a strong predictor of missed cervical cancer screenings.^52^ The decreased likelihood of nonsmokers missing breast and cervical cancer screenings have also been confirmed in other studies,^50^ and smoking is a known risk factor for breast and cervical cancer.^50,53^

Finally, studies have shown an association between women who are less educated and their likelihood of missing a cervical cancer screening, thereby revealing the disparities resulting from differences in socioeconomic status; those women who are less educated perhaps have less knowledge about these medical screenings and their ability to prevent cancer.^50,51^

Interpretation of these results has several limitations. First, the BVHA data are self-reported and thus prone to recall bias when answering several of the questions. Second, the survey did not ask for the reasons women missed screenings; therefore, we could only speculate on plausible reasons based on existing literature. Third, even after controlling for potential confounders, we could not rule out other underlying diseases that may have led to missed screening.

Finally, since the BVHA data are cross-sectional, we cannot generalize this to the whole population, nor can we establish long-term patterns. Despite these limitations, our study has several strengths. First, it adds to the existing literature by exploring the relationship between BMI and cancer screening. Second, our study contributes to the sparse literature on factors associated with cancer screening among semiurban populations; our sample size was large and representative of south-central Texas. Last, our study utilized community health assessment data, thus providing evidence of potential leverage points for increasing cancer screening rates in the Brazos Valley.

## Conclusion

This study found that BMI may be a weak but important predictor of cancer screening behavior for women, particularly for cervical cancer screening. Breast cancer (mammography) and cervical cancer (Pap test) screening are effective in reducing mortality and morbidity in women. Although breast and cervical cancer screening should be the objective for all women, additional screening efforts should be targeted toward women with higher BMI. Based on the increased risk of breast and cervical cancer for overweight and obese women, it is important that more women meet the recommended screening guidelines stipulated by USPHTF.

Additional research is needed to explore specific reasons why women do not get these vital screenings. Public health efforts should be directed at promoting breast and cervical cancer screening behavior among women who are obese or overweight. The findings of this present study have useful implications for policymakers, health care professionals, and researchers in designing and implementing efforts to promote breast and cervical cancer screenings in this population.

## Abbreviations Used

BMI: body mass index
BVHA: Brazos Valley Health Assessment
CI: confidence interval
hrHPV: high-risk human papillomavirus
OR: odds ratio
SD: standard deviation
USPSTF: U.S. Preventative Services Task Force

## References

[1] World Health Organization. Overweight and obesity. Geneva, Switzerland: World Health Organization, 2016. Available at: www.who.int/mediacentre/factsheets/fs311/en/ Accessed January 10, 2022.

[2] World Health Organization. Breast cancer: Prevention and control. Geneva, Switzerland: World Health Organization, 2017. Available at: www.who.int/cancer/detection/breastcancer/en/index1.html Accessed January 10, 2022.

[3] Siegel RL, Miller KD, Jemal A. Cancer statistics, 2016. CA Cancer J Clin 2016;66:7–30. 26742998 10.3322/caac.21332

[4] Kassebaum NJ, Bertozzi-Villa A, Coggeshall MS, et al. Global, regional, and national levels and causes of maternal mortality during 1990–2013: A systematic analysis for the Global Burden of Disease Study 2013. Lancet 2014;384:980–1004. 24797575 10.1016/S0140-6736(14)60696-6 PMC4255481

[5] Centers for Diseases Control and Prevention (CDC). Basic information about breast cancer. Atlanta, GA, 2016. Available at: https://www.cdc.gov/cancer/breast/basic_info/index.htm Accessed January 10, 2022.

[6] Centers for Diseases Control and Prevention (CDC). Cervical cancer statistics. Atlanta, GA, 2017. Available at: https://www.cdc.gov/cancer/cervical/statistics/ Accessed January 10, 2022.

[7] National Cancer Institute. Cancer screening. Bethesda, MD, 2017. Available at: https://www.cancer.gov/about-cancer/screening Accessed January 10, 2022.

[8] National Cancer Institute. Cancer screening overview (PDQ^®^)-Health professional version. Bethesda, MD, 2017. Available at: https://www.cancer.gov/about-cancer/screening/hp-screening-overview-pdq Accessed January 10, 2022.

[9] American Cancer Society. What are the key statistics about cervical cancer? Atlanta, GA, 2017. Available at: https://www.cancer.org/cancer/cervical-cancer/about/key-statistics.html Accessed January 10, 2022.

[10] U.S. Preventative Health Task Force. Cervical cancer: Screening. Rockville, MD, 2012. Available at: https://www.uspreventiveservicestaskforce.org/uspstf/recommendation/cervical-cancer-screening-2012 Accessed January 10, 2022.

[11] U.S. Preventative Health Task Force. Breast cancer: Screening. Rockville, MD, 2009. Available at: https://uspreventiveservicestaskforce.org/uspstf/recommendation/breast-cancer-screening-2009 Accessed January 10, 2022.

[12] Arnold M, Pandeya N, Byrnes G, et al. Global burden of cancer attributable to high body-mass index in 2012: A population-based study. Lancet Oncol 2015;16:36–46. 25467404 10.1016/S1470-2045(14)71123-4 PMC4314462

[13] Renehan AG, Zwahlen M, Egger M. Adiposity and cancer risk: New mechanistic insights from epidemiology. Nat Rev Cancer 2015;15:484. 26205341 10.1038/nrc3967

[14] Centers for Diseases Control and Prevention (CDC). About Adult BMI. Atlanta, GA, 2016. Available at: https://www.cdc.gov/healthyweight/assessing/bmi/adult_bmi/index.html#Why Accessed January 10, 2022.

[15] Harvey AE Lashinger, LM, Hursting SD. The growing challenge of obesity and cancer: An inflammatory issue. Ann NY Acad Sci 2011;1229:45–52. 21793838 10.1111/j.1749-6632.2011.06096.x

[16] Harvie M, Hooper L, Howell AH. Central obesity and breast cancer risk: A systematic review. Obes Rev 2003;4:157–173. 12916817 10.1046/j.1467-789x.2003.00108.x

[17] Flegal KM, Kruszon-Moran D, Carroll MD, Fryar CD, Ogden CL. Trends in obesity among adults in the United States, 2005 to 2014. JAMA 2016;315:2284–2291. 27272580 10.1001/jama.2016.6458 PMC11197437

[18] American Cancer Society. Obesity and cancer. Atlanta, GA, 2017. Available at: https://www.cancer.gov/about-cancer/causes-prevention/risk/obesity/obesity-fact-sheet Accessed January 10, 2022.

[19] Calle EE, Rodriguez C, Walker-Thurmond K, Thun MJ. Overweight, obesity, and mortality from cancer in a prospectively studied cohort of US adults. N Engl J Med 2003;348:1625–1638. 12711737 10.1056/NEJMoa021423

[20] Bianchini F, Kaaks R, Vainio H. Overweight, obesity, and cancer risk. Lancet Oncol 2002;3:565–574. 12217794 10.1016/s1470-2045(02)00849-5

[21] Munsell MF, Sprague BL, Berry DA, Chisholm G, Trentham-Dietz A. Body mass index and breast cancer risk according to postmenopausal estrogen-progestin use and hormone receptor status. Epidemiol Rev 2014;36:114–136. 24375928 10.1093/epirev/mxt010 PMC3873844

[22] American Cancer Society. Does body weight affect cancer risk? Atlanta, GA, 2017. Available at: https://www.cancer.org/cancer/cancer-causes/diet-physical-activity/body-weight-and-cancer-risk/effects.html Accessed January 10, 2022.

[23] Benedetto C, Salvagno F, Canuto EM, Gennarelli G. Obesity and female malignancies. Best Pract Res Clin Obst 2015;29:528–540. 10.1016/j.bpobgyn.2015.01.003 25779915

[24] Haakinson DJ, Leeds SG, Dueck AC, et al. The impact of obesity on breast cancer: A retrospective review. Ann Surg Oncol 2012;19:3012–3018. 22451232 10.1245/s10434-012-2320-8

[25] Conroy SM, Maskarinec G, Wilkens LR, White KK, Henderson B, Kolonel LN. Obesity and breast cancer survival in ethnically diverse postmenopausal women: The multiethnic cohort study. Breast Cancer Res Treat 2011;129:565–574. 21499688 10.1007/s10549-011-1468-4 PMC3164157

[26] Poorolajal J, Jenabi E. The association between BMI and cervical cancer risk: A meta-analysis. Eur J Cancer Prev 2016;25:232–238. 25932869 10.1097/CEJ.0000000000000164

[27] American Cancer Society. What are the risk factors for cervical cancer? Atlanta, GA, 2017. Available at: https://www.cancer.org/cancer/cervical-cancer/causes-risks-prevention/risk-factors.html Accessed January 10, 2022.

[28] Ludman EJ, Ichikawa LE, Simon GE, et al. Breast and cervical cancer screening: Specific effects of depression and obesity. Am J Prev Med 2010;38:303–310. 20171532 10.1016/j.amepre.2009.10.039 PMC2835516

[29] Ferrante JM, Chen PH, Jacobs A. Breast and cervical cancer screening in obese minority women. J Womens Health 2006;15:531–541. 10.1089/jwh.2006.15.531 16796480

[30] Maruthur NM, Bolen SD, Brancati FL, Clark JM. The association of obesity and cervical cancer screening: A systematic review and meta-analysis. Obesity 2009;17:375–381. 18997682 10.1038/oby.2008.480 PMC3008358

[31] Wee CC, McCarthy EP, Davis RB, Phillips RS. Screening for cervical and breast cancer: Is obesity an unrecognized barrier to preventive care? Ann Intern Med 2000;132:697–704. 10787362 10.7326/0003-4819-132-9-200005020-00003

[32] Cohen SS, Palmieri RT, Nyante SJ, et al. A review. Cancer 2008;112:1892–1904. 18361400 10.1002/cncr.23408

[33] Tekkel M, Veideman T, Rahu M. Use of mammography, Pap test and prostate examination by body mass index during the developmental period of cancer screening in Estonia. Public Health 2011;125:697–703. 21907366 10.1016/j.puhe.2011.06.013

[34] Kim BH, BaekKoh S, Hur HK, Park JK, Park SM. Women's cancer screening according to body mass index in a cohort of rural Korean women. J Korean Acad Nurs 2009;39. 10.4040/jkan.2009.39.5.641 19901494

[35] Choi KH, Heo J, Kim S, Jeon YJ, Oh M. Factors associated with breast and cervical cancer screening in Korea: Data from a national community health survey. Asia-Pac J Public Health 2013;25:476–486. 24151045 10.1177/1010539513506601

[36] Park JK, Park HA, Park JJ, Cho YG. Obesity and screening compliance for breast and cervical cancer in Korean women. Asian Pac J Cancer Prev 2012;13:3271–3274. 22994746 10.7314/apjcp.2012.13.7.3271

[37] Fontaine KR, Heo M, Allison DB. Body weight and cancer screening among women. J Women Health Gen-B 2001;10:463–470. 10.1089/152460901300233939 11445045

[38] Wee CC, Phillips RS, McCarthy EP. BMI and cervical cancer screening among White, African-American, and Hispanic women in the United States. Obes Res 2005;13:1275–1280. 16076999 10.1038/oby.2005.152

[39] Bean-Mayberry B, Bastian L, Trentalange M, et al. Associations between provider designation and female-specific cancer screening in women veterans. Med Care 2015;53:S47–S54. 25767975 10.1097/MLR.0000000000000323 PMC5477654

[40] Yancy WS, McDuffie JR, Stechuchak KM, et al. Obesity and receipt of clinical preventive services in veterans. Obesity 2010;18:1827–1835. 20203629 10.1038/oby.2010.40

[41] Prochaska JD, Sharkey JR, Ory MG, Burdine JN. Assessing healthful eating among community dwelling rural older adults using self-reported fruit and vegetable consumption via a community-wide mail-out health status assessment. J Nutr Elder 2006;25:101–112. 10.1300/j052v25n02_07 17182469

[42] Mitchell RS, Padwal RS, Chuck AW, et al. Cancer screening among the overweight and obese in Canada. Am J Prev Med 2008;35:127–132. 18617081 10.1016/j.amepre.2008.03.031

[43] Simoes EJ, Newschaffer CJ, Hagdrup N, et al. Predictors of compliance with recommended cervical cancer screening schedule: A population-based study. J Community Health 1999;24:115–130. 10202691 10.1023/a:1018754307718

[44] Littman AJ, Koepsell TD, Forsberg CW, et al. Preventive care in relation to obesity: An analysis of a large, national survey. Am J Prev Med 2011;41:465–472. 22011416 10.1016/j.amepre.2011.07.020

[45] Altman DG, Royston P. The cost of dichotomising continuous variables. BMJ 2006;332:1080. 16675816 10.1136/bmj.332.7549.1080 PMC1458573

[46] Burhansstipanov L, Dresser CM. Documentation of the cancer research needs of American Indians and Alaska Natives (Native American Monograph No. 1). Bethesda, MD: National Cancer Institute, 1993.

[47] May DS, Lee NC, Nadel MR, Henson RM, Miller DS. The National Breast and Cervical Cancer Early Detection Program: Report on the first 4 years of mammography provided to medically underserved women. Am J Roentgenol 1998;170:97–104. 9423608 10.2214/ajr.170.1.9423608

[48] Henson RM, Wyatt SW, Lee NC. The National Breast and Cervical Cancer Early Detection Program: A comprehensive public health response to two major health issues for women. J Public Health Manag 1996;2:36–47. 10186667

[49] Abdou CM, Fingerhut AW. Stereotype threat among black and white women in health care settings. Cultur Divers Ethnic Minor Psychol 2014;20:316–323. 25045944 10.1037/a0036946 PMC5449200

[50] Amonkar MM, Madhavan S. Compliance rates and predictors of cancer screening recommendations among Appalachian women. J Health Care Poor Underserved 2002;13:443–460. 12407962 10.1353/hpu.2010.0582

[51] Calle EE, Flanders WD, Thun, MJ, Martin LM. Demographic predictors of mammography and Pap smear screening in US women. Am J Public Health 1993;83:53–60. 8417607 10.2105/ajph.83.1.53 PMC1694510

[52] Østbye T, Taylor DH, Yancy WS, Krause KM. Associations between obesity and receipt of screening mammography, Papanicolaou tests, and influenza vaccination: Results from the health and retirement study (HRS) and the asset and health dynamics among the oldest old (AHEAD) study. Am J Public Health 2005;95:1623–1630. 16051935 10.2105/AJPH.2004.047803 PMC1449407

[53] Johnson KC, Hu J, Mao Y. Passive and active smoking and breast cancer risk in Canada, 1994–1997. Cancer Cause Control 2000;11:211–221. 10.1023/a:1008906105790 10782655

